# Clinicians’ perspectives and perceived barriers to caring for patients with alcohol use disorder and cirrhosis

**DOI:** 10.1186/s13722-022-00292-8

**Published:** 2022-02-09

**Authors:** Emily Johnson, Sumantra Monty Ghosh, Vijay John Daniels, T. Cameron Wild, Puneeta Tandon, Ashley Hyde

**Affiliations:** 1 Division of Gastroenterology (Liver Unit), Department of Medicine, University of Alberta, Edmonton, AB Canada; 2 Division of General Internal Medicine, Department of Medicine, University of Alberta, Edmonton, AB Canada; 3 School of Public Health, University of Alberta, Edmonton, AB Canada

**Keywords:** Alcohol use disorder, Cirrhosis, Alcohol-associated liver disease, Clinician perspectives, Interviews

## Abstract

**Background:**

Alcohol use disorder (AUD) is one of the leading etiologies for liver cirrhosis and liver transplantation. Few individuals with AUD receive guideline-based care in the form of screening, brief intervention, referral to treatment, or prescription of pharmacotherapy for relapse prevention. We interviewed clinicians across Alberta to assess the current experience and perceived barriers to managing AUD in people who have cirrhosis. The aim of this paper is to summarize these findings to inform the development of an educational intervention.

**Methods:**

We used a qualitative descriptive approach to explore the experiences of clinicians who care for patients with cirrhosis and AUD in Alberta. We conducted semi-structured interviews directed by an interview guide. Interviews were recorded and transcribed verbatim. We used an inductive thematic analysis approach whereby transcripts were coded, with codes grouped into larger categories, then themes.

**Results:**

Sixteen clinicians participated in this study. Many participants acknowledged that they do not use a standardized approach to screening, brief intervention, and referral to treatment. Through thematic analysis we identified four themes surrounding barriers to managing AUD in patients with cirrhosis: (i) Practicing within knowledge constraints, (ii) Navigating limited resources and system challenges, (iii) Balancing the complexity of cirrhosis and AUD, and (iv) Acknowledging the influence of provider perceptions on care.

**Conclusion:**

This article presents the perspectives of clinicians who care for people who have AUD and cirrhosis. Significant barriers exist, including limited knowledge and resources, systemic challenges, and patient complexity. The information gathered will be used to develop an educational intervention that will delve deeper into these issues in order to have the greatest impact on clinicians who routinely interface with this patient population.

## Background

Alcohol use disorder (AUD) is a chronic, relapsing condition that affects approximately 14% of individuals in North America [1, 2]. AUD can lead to a host of negative health consequences, with one of the most prevalent being alcohol-associated cirrhosis [3, 4]. Individuals with concomitant cirrhosis and AUD are at increased risk of experiencing adverse health consequences attributable to their liver disease, in addition to psychological stress, financial hardship, and homelessness [5]. Despite these negative consequences, and the evidence to support the benefit of AUD related treatment [6, 7], less than 20% of individuals with AUD receive psychological/behavioural therapy with or without pharmacotherapy for relapse prevention [1]. While current statistics are lacking for Canada, a recent study in the United States found that among 21,270 adults with AUD, only 5.8% reported receiving treatment [8].

Recent clinical practice guidelines from the American Association for the Study of Liver Disease (AASLD) and the European Association for the Study of the Liver (EASL) have stressed the importance of considering not only the management of direct liver complications (e.g. alcohol associated hepatitis), but also intervening to address the root cause of the liver disease—alcohol consumption [9, 10]. For patients who meet criteria for AUD and/or have alcohol-related liver disease, a multidisciplinary integrated care approach including the involvement of an addiction medicine specialist and initiation of pharmacotherapy for relapse prevention, is recommended [9, 10]. Current evidence supports the use of Acamprosate, as well as the off-label use of Baclofen and Gabapentin for relapse prevention in people who have liver disease [9, 11]. Though an integrated multidisciplinary care approach is the recognized gold standard for patients with concomitant AUD and cirrhosis, integrated clinics and addiction medicine specialists are not easily accessible to many clinicians who care for patients with cirrhosis [12]. Indeed, there are no clinics in our province where cirrhosis care and AUD care are integrated. An interim step to support clinicians without access to an integrated care model would be to increase provider confidence with skills including screening, brief intervention, referral to psychosocial/behavioral therapy (SBIRT), and confidence with initiating pharmacotherapy for relapse prevention [13].

The multiple treatment options for AUD range from behavioural therapy (Cognitive behavioural therapy (CBT), motivational enhancement therapy (MET)), psychological support (counselling, trauma support), mutual aid fellowships (Alcoholics Anonymous (AA)) provided alone or, ideally, in combination with pharmacotherapy for relapse prevention [14, 15]. Taken in conjunction with patient wishes to change their alcohol consumption, these treatment options may help to improve the likelihood of abstinence and reduce the risk of alcohol-related complications. There is evidence to support the effectiveness of AUD treatment among people with liver disease [14, 15], but these treatments are greatly underused [1] despite frequent interactions with the healthcare system.

Individuals with AUD can present with liver related complications, or additional physical and mental health conditions, such as depression and post-traumatic stress disorder [16]. Each of these point-of-care interactions represents an opportunity for clinicians (e.g., primary care, emergency medicine, hepatology and hospitalist, nurse practitioners etc.) to intervene and support patients to reduce or cease their hazardous consumption of alcohol. Low rates of SBIRT implementation [17] lead to delays in treatment and worse mental and physical health outcomes [18]. By increasing SBIRT practices and prescription of pharmacotherapies for relapse prevention, more patients may have the opportunity to connect to treatment. After implementation of SBIRT practices in emergency departments for example, a systematic review by Barata et al. demonstrated fewer alcohol related consequences and repeat visits [19]. Hays et al. reported that implementation of SBIRT practices into their trauma center resulted in significant increases in patient acceptance of referral to an outpatient treatment center for their substance use disorder [20]. In order to change practice habits and reduce the burden of AUD, it is crucial that we understand the barriers to SBIRT and initiation of pharmacotherapies for relapse prevention in cirrhosis related AUD care.

To date, there have only been two studies that have explored clinicians’ experiences in managing AUD in patients with cirrhosis [21, 22]. Using a survey-based approach, key findings have included the lack of adoption of a standardized approach to AUD management, and low reported knowledge and comfort around pharmacotherapy for relapse prevention. Survey-based approaches are limited by the decision of which items to include. This is unlike qualitative methodology which can include open-ended questions and the ability to ask participants to expand on their experiences and perspectives. Moreover, clinicians in existing survey-based studies have primarily been from a gastroenterology/hepatology background, with no representation of other sub-specialties who also play a vital role in the circle of care (e.g., primary care, internal medicine, emergency medicine). To our knowledge, we are unaware of a qualitative exploration of barriers and facilitators around AUD management in people with cirrhosis involving primary care providers and gastroenterologists/hepatologists.

To address these knowledge gaps, the goal of this study was to describe the experience of clinicians caring for patients with concomitant AUD and cirrhosis. Specifically, we wanted to answer the following research questions: (i) What is the experience and present practices of clinicians caring for patients with cirrhosis and AUD, and (ii) What are the perceived barriers to providing care to patients with cirrhosis and AUD?

## Methods

We used a qualitative descriptive approach [23] to address the research objectives. This study was approved by the Health Ethics Research Board at the University of Alberta (Pro00089501). Informed consent was obtained prior to commencing interviews with participants.

### Sampling and recruitment

The present study is part of Cirrhosis Care Alberta (CCAB) a multi-site pragmatic trial aimed at improving the quality of care for patients with cirrhosis [24]. A portion of this trial is devoted to improving care for patients with cirrhosis and AUD through implementation of a standardized screening, intervention and treatment approach including the prescription of pharmacotherapy for relapse prevention. Participants were employed at hospital sites involved in the parent trial and were invited to participate in the present study by the principal investigator (PT) or additional CCAB site leads via email. A total of 19 participants were invited to join the study from 8 hospital sites (5 urban hospitals, 3 community hospitals) and practice settings that included gastroenterology/hepatology, internal medicine, and primary care; 3 did not wish to participate due to time constraints. We used a purposive sampling strategy to guide selection of participants to ensure that the study participants represented diverse professional roles (physician vs. nurse practitioner), geographic location of practice (North Zone, South Zone, Central Zone, Edmonton Zone and Calgary Zone), years of practice, and urban and community hospital sites.

### Data collection

Semi-structured interviews [25] were conducted by the first author (EJ) of this paper and supervised by a team member (AH) with expertise in qualitative methodology. Interviews were conducted via Zoom from January 2021–March 2021 and lasted 10–46 min (mean = 25 min). Development of the interview guide by EJ and AH (Table 1) was informed by a desire to explore current AUD management practices and perspectives of clinicians in Alberta caring for patients with AUD and cirrhosis. Interviews were audio recorded and transcribed verbatim. At the outset of each interview, demographic data were collected and following each interview, field notes detailing the interviewer’s preliminary impressions were recorded. The interview guide was refined as interviews progressed to probe areas of interest and emerging themes. Interviews progressed until saturation was achieved, with no new concepts emerging after 16 interviews.

**Table 1:** Semi-structured interview guide

1. Can you tell me about your experiences with caring for patients who have AUD and cirrhosis?
2. What are your challenges in providing care for patients with AUD and cirrhosis?
3. What resources do you feel are missing for you to be able to provide optimal care to patients with AUD and cirrhosis?
4. Can you tell me about how allied health (particularly social work and addictions) are involved in the care of a patient with AUD and cirrhosis at your site?
5. Can you tell me about what kind of educational resources would enhance your practice when caring for patients with AUD and cirrhosis?
6. Can you tell me how caring for patients with cirrhosis and AUD has changed since the start of the COVID-19 pandemic?

### Data analysis

We used a theoretical thematic analysis approach [26] whereby codes and themes were identified in an inductive manner based on a desire to understand our participant’s experience of caring for patients who have AUD and cirrhosis. Analyses were conducted by two members of the research team (EJ and AH) who coded several transcripts separately, then came together to develop a coding framework based on consensus. Following development of the framework, the remainder of the transcripts were coded by EJ. Frequent meetings with our team member with qualitative expertise (AH) and principal investigator (PT) enabled verification of emerging categories and themes. An audit trail detailing methodological and analytical decisions was maintained. NVIVO Pro (Version 12) was used for data management and to facilitate analysis [27].

## Results

**Fig. 1:**
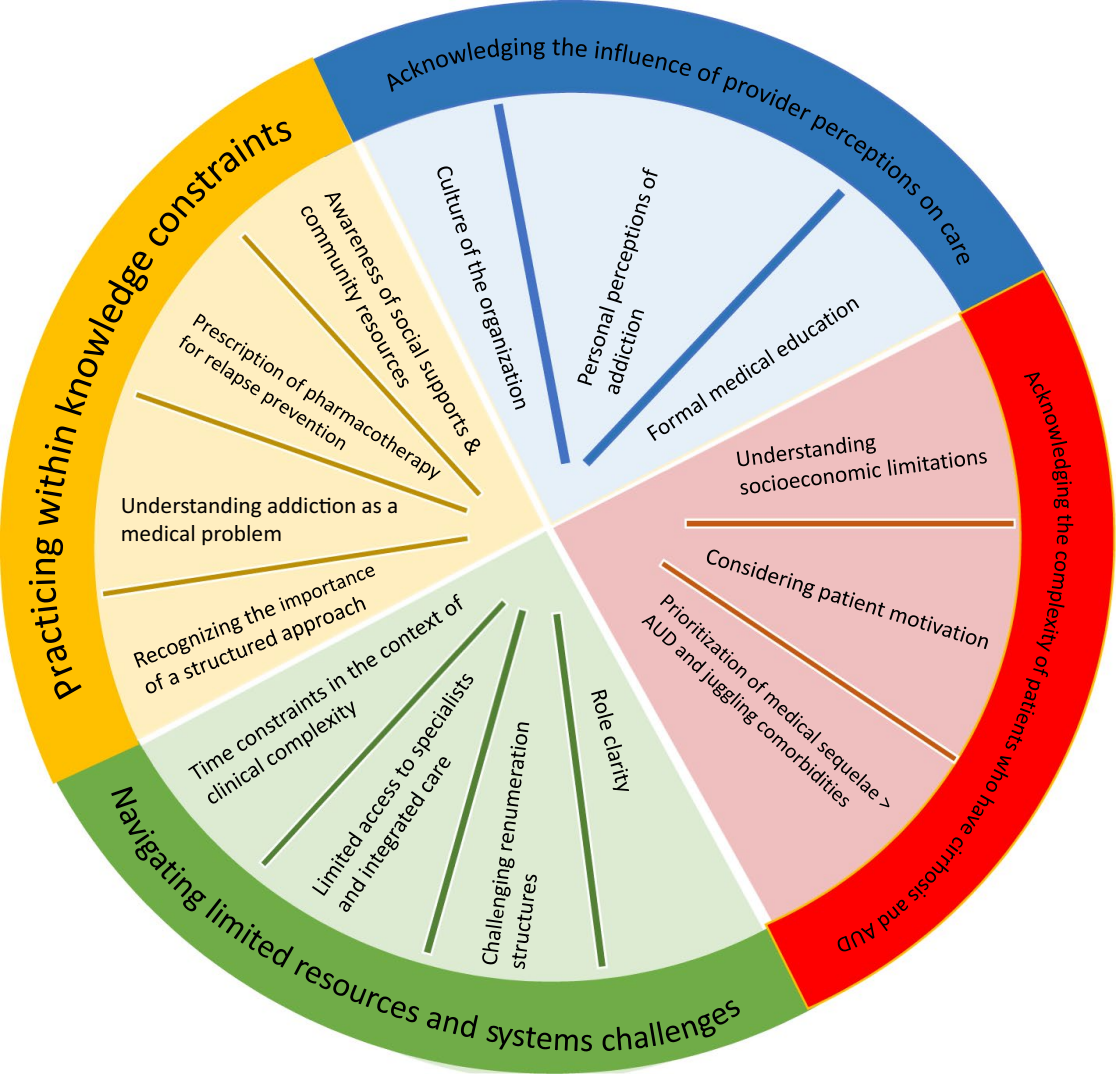
Major themes and codes

We conducted a total of 16 interviews with 11 physicians and 5 nurse practitioners. The majority (n = 12) were specialized in gastroenterology and hepatology, and the remainder were specialized in primary care (3) and internal medicine (1). Participants were recruited from all five Alberta Health Services (AHS) care delivery zones and both sexes were equally represented (50% male, 50% female). Additional demographic details are presented in Table 2. Four key themes emerged that described the experience of providing care to patients with cirrhosis and AUD: (i) Practicing within knowledge constraints, (ii) Navigating limited resources and system challenges, (iii) Balancing the complexity of cirrhosis and AUD, and (iv) Acknowledging the influence of provider perceptions on care (Results are presented graphically in Fig. 1).

**Table 2:** Demographic characteristics

Variable	N (%)
Gender	
Male	8 (50)
Female	8 (50)
Practice Specialization	
Family Doctor	3 (19)
Gastroenterologist/Hepatologist	12 (75)
Internist	1 (6)
Professional role	
Medical doctor (MD)	11 (69)
Nurse Practitioner (NP)	5 (31)
Practice Zone (Alberta Health Services)	
North Zone	2 (12.5)
Edmonton Zone	8 (50)
Central Zone	2 (12.5)
Calgary Zone	3 (19)
South Zone	1 (6)

### Theme #1: Practicing within knowledge constraints

Nearly all participants spoke about their limited knowledge of how to provide treatment for AUD in people who have cirrhosis. This perceived “lack of understanding” and knowledge stemmed from an absence of formal training and familiarity with treatment options for this patient population. One participant shared, “…we were never really educated about it [AUD] or didn't really know what the options were”. Though participants described their familiarity with clinical practice guidelines for management of cirrhosis, many did not use guidelines for AUD, instead relying on their ‘gut instincts’ and ‘spidey senses’ to assess their patient’s alcohol use and determine whether it required clinical intervention. Of this, one participant said,

*Usually, you get a fairly good idea about how much alcohol [a patient] uses…depending on if they tell me if they drink two beers a week for years, I don’t usually explore it much further.* (Participant 12, MD, Hepatology)

Further, participants did not routinely employ a validated screening measure to assess for AUD or use a structured approach to brief intervention. For some, this approach was attributed to being unaware, while for others it was due to a perceived lack of clinical evidence including the “limited research done on this patient population” and concerns over “how effective” various interventions are in this group of patients. For example, one participant noted:

*I don’t go through a whole questionnaire or anything like that…if I know that they’re already an alcoholic, I don’t really think that I need to screen them for the severity because I don’t really treat them differently.* (Participant 9, MD, Gastroenterology/Hepatology)

This unstructured approach extended to the process of diagnosing AUD. Participants stated that they typically did not use a set of diagnostic criteria such as those in the Diagnostic and Statistical Manual of Mental Disorders, 5th Edition (DSM-5) because they “believe their patients would meet the criteria anyway” and that “it doesn’t take much to make the diagnosis” especially with a “collateral history” such as the presence of cirrhosis. One participant reflected on their experience with diagnosing a patient with AUD when they were unfamiliar with the patient’s history.

*I personally have a hard time ascribing a diagnosis to fit to symptoms that I didn’t personally observe…you’re going to start giving people diagnoses that they never actually had and altering the way they interact with the healthcare system.* (Participant 1, MD, Primary Care).

Participants also shared their discomfort with the prescription of pharmacotherapy for relapse prevention as a result of their perceived gaps in knowledge. They noted that the advances in addiction medicine contributed to their knowledge deficits: “a lot of these medications came out after I finished training…”. Other participants reflected on how clinical norms in treating AUD combined with their lack of knowledge:

*In all the settings that I worked, [prescribing pharmacotherapy] is just not something we do. And I realized that it is a knowledge deficit for all of us and we need to get better at that.* (Participant 5, NP, Gastroenterology/Hepatology).

Several participants shared that they “did not feel comfortable” prescribing pharmacotherapy for relapse prevention for AUD in patients who also had cirrhosis due to what they see as limited “viable options” resulting from a paucity of randomized controlled trials testing the efficacy and safety in this unique population. Further, they also reported feeling generally unaware of community resources to compliment potential medical therapies: “I don’t even know where it [addictions clinic] is…I don’t know what’s offered there”. One participant reflected on how these knowledge constraints could ultimately impact the patient’s desire to receive treatment for AUD:

*If I knew a bit more, I’d feel more confident. And if people [clinicians] are more confident in what they’re recommending, generally their patients are more interested in it”* (Participant 1, MD, Primary Care Provider)

### Theme #2: Navigating limited resources and systems challenges

Many participants spoke of the resource challenges they encountered in their care of patients with cirrhosis and AUD. For some, the most striking resource limitation was their own clinical time, which they felt prevented them from providing “reliable follow up.” They recognized that effectively treating patients with concomitant cirrhosis and AUD required a significant investment of time:

*The trouble is we just don’t have 45 minutes to sit down with a patient and go over everything and tell them exactly what to do.* (Participant 2, MD, Gastroenterology/Hepatology)

For clinicians that practiced in an acute care setting, this notion of time as a limited resource was especially pronounced. One participant said the following regarding building relationships with patients admitted to acute care units:

*It is a challenge in acute care because we only see them for such a short snippet of time. We can’t build that relationship, follow that relationship through, and see it to the end. We’re really trying to put a Band-Aid on something that needs an abdominal pad because we see them for such a short time period.* (Participant 13, NP, Gastroenterology/Hepatology).

This sentiment was echoed by those in specialist settings who expressed concerns over providing reliable timely follow up in the context of pharmacotherapy for relapse prevention:

*It’s not really a drug I want to be prescribing myself from a practice management standpoint…because if there is an issue, they’re going to be able to see their primary care provider way easier than seeing me just by volume and access.* (Participant 12, MD, Gastroenterology/Hepatology)

Struggles with “workload management” were also found to impede participant’s ability to provide the additional supports they deemed necessary for their patients. They noted that coordinating psychosocial services was difficult to achieve in a timely manner, with one participant saying, “it’s actually a disservice because it’s hard to target therapy because of that.” Another participant noted that medications for relapse prevention “don’t work on their own” and interventions like “cognitive behavioural therapy” are important to achieving the best outcome in patients but “getting both of those pieces coordinated together” was a “challenge.”

Participants also felt that a shortage of manpower affected their care of patients with cirrhosis and AUD. Participants who worked as PCPs in the community spoke about the perceived inability to refer patients to see a liver specialist as they “wouldn’t see just anyone due to the sheer volume of patients”. This extended to a perceived lack of addiction medicine support. One participant stated:

*I don’t really have anybody accessible to me that I can say to my patient, ‘oh this person is willing to see you urgently.’ It’s really hard for them to get in to see somebody.* (Participant 4, MD, Gastroenterology/Hepatology)

Similarly, another participant acknowledged how providing care in a rural area further limited the support they could provide:

*Endemic to my zone is the sense that it’s very poorly funded and it lives between two giant polarities. And when you don’t have the tools, you often become really resistant to opening those doors anyways, because you feel like you don’t know where to go with it, it just becomes overwhelming.* (Participant 3, MD, Gastroenterology/Hepatology).

Others who provided care for individuals in rural settings further expressed that limited services in these communities makes it challenging to access addiction medicine in particular. One participant commented:

*We service a large population outside the city, so being able to do outreach to the different facilities or different cities or towns themselves would be helpful. But it’s challenging with only a limited number of resources.* (Participant 4, MD, Gastroenterology/Hepatology).

Participants also described systems challenges including role clarity and the confusion about which provider should initiate and maintain AUD treatment for patients with cirrhosis. Several participants who practiced as liver specialists felt that treatment was beyond their role as “the alcohol use is well established” by the time they see the patients. This sentiment was similar regarding prescription of pharmacotherapy for relapse prevention, with most liver specialists indicating that they were not comfortable initiating them because “it’s very hard to see these patients for follow up in three to four months’ time”. They felt that their inability to provide timely follow up made it risky for patient’s overall health if they experienced an “issue with the medication”. Several specialists did, however, indicate that they would be inclined to initiate pharmacotherapy for relapse prevention “if [a patient] is in a steady state and the family doctor can continue the refills”.

For most PCPs, these perceptions about whose responsibility it is to care for patients with AUD and cirrhosis, led to mixed feelings regarding their role. One PCP said the following about feeling equipped to support patients with AUD and cirrhosis:

*Part of me thinks like in a perfect world, there’d be some sort of place where I’d send them and somebody else would deal with them. But that’s not fair because as a primary care practitioner, we know in the evidence that the very best place they receive care is with their family doctor at a place that they feel comfortable. So, though I would love to be able to pawn someone out, that’s not fair and that wouldn’t be in my patient’s best interest.* (Participant 10, MD, Primary Care).

Deciding who is responsible for caring for patients with AUD and cirrhosis was an issue identified by a number of participants. Division of liver care from the AUD care was described by specialists and PCPs, with many acknowledging time constraints with specialists as a major factor, but realizing many things get “dumped” onto primary care to manage.

### Theme #3: Balancing the complexity of cirrhosis and AUD

Apart from identifying personal and system-level barriers to effectively caring for patients with AUD and cirrhosis, participants also discussed challenges in the concurrent management of the conditions. Multiple participants described the often-high acuity of patients with concomitant cirrhosis and AUD and how this impacted their approach to care:

*I think one of the biggest hurdles is that these patients are quite sick, and their body is more fragile, and so these hits that normally people would bounce back from, they just take longer to bounce back from.* (Participant 1, MD, Primary Care).

Similarly, another participant shared “I’m just trying to deal with the biggest thing” with AUD “sort of on the backburner, it’s like when you feel better, we can get you on that”. They acknowledged that this acuity also influenced their willingness to prescribe pharmacotherapy for relapse prevention. One participant shared:

*A lot of my patients, when they wind up seeing me in the clinic, have very advanced cirrhosis…where my choices are very, very limited. I don’t see a lot of people now that are Child Pugh A [i.e., the absence of liver related complications] …it’s just sort of the way the practice is.* (Participant 12, MD, Gastroenterology/Hepatology).

Participants not only discussed the need for consideration of the severity of the patient’s cirrhosis when planning treatment, but also the consideration of the severity of AUD. For patients with severe AUD, care was perceived as more challenging with one participant stating:

*The biggest challenge in some of these folks is, it’s not even the use, I can handle the use piece…it’s just the behavioural piece, which makes lives chaotic and makes it hard to relate to people and makes them sometimes unreliable and just makes them fragile and their health very precarious. And they fall, and they fall back down into a dark place, a tunnel, a place that I can’t find them. And then they show up in hospital and I haven’t been able to track them down for six or eight months…. I think that’s the biggest challenge.* (Participant 3, MD, Gastroenterology/Hepatology)

Participants also shared how “patient motivation” and “initiative” served as an impetus for initiating treatment. One participant reflected on the importance of patient responsibility and how “it sort of establishes itself really quickly, those that are going to be motivated and self-starters”. Others considered patient “compliance” with lab tests, appointments, and previous medication as indicative of that patient’s eligibility to receive treatment for their AUD. However, participants also expressed the difficulty of living with AUD, remembering “it is an illness” and it “is not easy” to maintain abstinence. One participant reflected on this balance between understanding and being honest with their patient regarding their alcohol use:

*Being empathetic is one thing but we can’t be delusional in thinking that we can massage the reality for our patients. Say if I tell you that alcohol is not the problem, maybe they will change. No, we have to be honest with our patients.* (Participant 14, MD, Primary Care).

### Theme # 4: Acknowledging the influence of provider perceptions on care

Participants approach to caring for patients with AUD and cirrhosis was influenced by their formal medical education, personal perceptions of addiction, and culture of the organizations in which they practiced. Participants acknowledged how “massively stigmatized” liver disease was, with AUD tending to compound this stigma. One participant noted that “there’s a lot of prejudice among physicians about these conditions” adding that the burden can feel worse for patients “who must carry that stigma with them.”

Several participants also spoke about stigmatized attitudes toward patients with cirrhosis because of their alcohol dependence and perceived ‘worthiness’ of those patients for other services. They believed that these attitudes would lead to a cascade of events in which future encounters a patient had with the healthcare system would be colored by judgement from other healthcare providers. One physician reflected on how this affected the patient’s journey through the healthcare system:

*There’s a lot of assumptions [about patients with AUD and cirrhosis]-they’re not a transplant candidate, they’re not an ICU candidate, they’re not going to follow-up, so what’s the point in giving recommendations? It’s very, very disheartening.* (Participant 9, Gastroenterology/Hepatology)

While personal perceptions and organizational culture influenced care of patients with AUD and cirrhosis, participants also noted differences in care according to the career stage of the clinician. This not only affected willingness to use of pharmacotherapies for relapse prevention, but also colored their interactions with patients and their understanding of AUD as a “brain-body” disease. One physician who recently completed their training commented on this, saying:

*I think it’s a generational thing as well, this concept of using agonist therapies or partial agonist therapies like anti-craving meds, wasn’t really something that was done even before the opioid crisis…you had to have a license to put people on suboxone. And the same thing happens with other substance use disorders. People tend to continue to practice the way they were trained, and the way most people were trained was heavily focused on abstinence.* (Participant 1, MD, Primary Care)

This was reinforced by a provider who was in the later stages of their medical career, who acknowledged the evolving nature of caring for patients with AUD and the field of addiction, saying “it has come a long way.” Although participants appreciated the influence of the “social aspects” of AUD like poverty, unemployment, and trauma, they acknowledged the tendency for most clinicians to “deal with [AUD] in a very medical way.”

## Discussion

Though there have been several studies of clinician practices, knowledge, and attitudes towards patients with cirrhosis and AUD [21, 22], to our knowledge our study is the first to qualitatively explore the experiences of clinicians working with this group of patients. The participants in our study described a number of challenges, and recognized the importance of providing person-centered, continuous care considerate of the medical complexity, motivation, and support systems available to the patient. Participants acknowledged that managing AUD and cirrhosis requires an understanding of the nuances of both conditions and the processes and factors that influence them. However, limited training, research and understanding of the physiological processes of AUD and its relevance to cirrhosis complications adds complexity which, for many of our participants, made it harder to manage either condition. Awareness of existing stigma by healthcare providers and the medical system were also brought forth by several participants as a factor preventing effective care of people with concomitant AUD and cirrhosis. Acknowledging the pervasiveness of stigma, readers will note that even within this manuscript, some quotes reflect disempowering language and attitudes.

A prominent finding of this study was the relative lack of training clinicians receive about caring for patients with AUD. This is congruent with data suggesting that less than 16% of clinicians report receiving adequate addiction medicine training either in medical school [28] or in their fellowship programs [21]. A report by the National Centre for Addiction and Substance Abuse at Columbia University has advocated for more addiction training “at every level—in medical school, residency training, continuing education and in practice” to prepare current and future clinicians to deal with all aspects of substance use disorder management [29].

Clinicians not surprisingly described feeling ill-equipped to employ standardized screening tools and brief interventions in their treatment for patients with AUD. Despite well-established clinical practice guidelines [9, 10, 30] that suggest screening, brief intervention, referral to treatment [31] and prescription of pharmacotherapy for relapse prevention, few clinicians in our study described using this approach. These findings align with previous research that found that less than 40% of clinicians use a validated measure to screen for AUD [32]. With a high specificity and sensitivity to identifying AUD, validated screening tools [33] are important tools to avoid missing patients with milder use and falsely diagnosing a patient with AUD, a mistake that can occur when using clinical intuition alone [34, 35]. In a meta-analysis by Mitchell et al. clinical judgement resulted in the incorrect diagnosis of AUD in 50% and 60% and of patients among hospital staff and PCPs, respectively [35]. A structured approach to brief interventions can reduce alcohol consumption [36]. At a systems-level, this can be supported by the integration of validated screening measures into electronic medical record systems. This increased screening rates for AUD (73.9%) [37] compared with the typical population (~ 25% in some data captured in the United States) [17]. Similar findings were observed with integrated reminders to conduct a brief intervention for patients with excess alcohol use [38].

Similar to screening and brief interventions, research also supports the effectiveness of pharmacotherapy for relapse prevention in reducing hepatic decompensation and long-term mortality in patients with cirrhosis and AUD [15, 39–44]. Both specialists and PCPs in our study shared their hesitancy in prescribing these medications as they had concerns with their inability to effectively monitor patient progress due to short appointment times with long waitlists for follow-up. This was consistent with other studies, which reported a short appointment window and inability to maintain follow up as the biggest hurdles to providing substance use disorder care [45–48]. This hesitancy was further compounded by the perceived lack of availability of addiction medicine services that could enable long-term follow up with a greater focus on the unique medical and psychological needs of these patients.

Clinicians in our study acknowledged that for many patients, AUD was the root cause of their medical comorbidities like cirrhosis. However, they described prioritizing treatment of medical sequelae like gastrointestinal bleeding, ascites, and hepatic encephalopathy over AUD, as those most significantly affected patient acuity. Indeed, some shared their hesitancy to initiate treatment for AUD unless patients were compliant with treatments for their comorbidities, like routine lab testing and lactulose therapy for hepatic encephalopathy. Several clinicians, both PCPs and specialists, expressed uncertainty about who should initiate treatment for the AUD. Specialists shared concerns about initiating treatment as they felt that due to long waitlists they could not provide timely follow up to monitor progress with prescribed AUD treatments. PCPs shared that they were often hesitant to initiate treatment because of the patient’s medical complexity, particularly their impaired liver function and tolerance of pharmacotherapeutics for AUD. Issues of role clarity, particularly who should initiate treatment for AUD, remain relatively unexplored in the literature. While there are several studies that explore the roles of PCPs and specialists who care for patients with cirrhosis and AUD, we were unable to identify any that specifically explored issues of role clarity in concomitant cirrhosis and AUD [43–45]. Given the increasing prevalence of AUD and cirrhosis, further exploration and explication of clinician roles is warranted.

Stigma from healthcare providers has been found to significantly contribute to negative perceptions of people with substance use disorders and sub-optimal care [49, 50]. Although several participants acknowledged existing personal and systems-level stigma towards individuals who have substance use disorders (including AUD), the views expressed in the interviews suggest significant work needs to be done to reduce stigma and improve outcomes and experiences for people affected by substance use disorders. Stigmatized language used to describe the pathophysiology of AUD (e.g. “revolving door”), people with AUD (e.g. “alcoholics”), and treatments were not empowering.

The majority of the clinicians we interviewed practiced in an urban setting with access to a tertiary care center with specialized gastroenterology and addiction medicine services. Despite this proximity, they expressed challenges in accessing these specialized services, including a lack of integration between these services and perceived paucity of addiction medicine specialists. This was amplified for clinicians who practiced in rural settings who noted limited access to specialized care, including behavioral therapy. These findings are congruent with the literature which notes significant differences in the treatment of patients for AUD in rural and urban settings [12, 51–54]. Though it may not be feasible to increase rural access to supports for patients with AUD, an increased awareness and visibility of processes to access these supports for both specialists and PCPs will no doubt benefit patients with AUD and cirrhosis.

## Limitations

Our study has several limitations. First, the participants of this qualitative study were clinicians recruited via convenience sampling based on their involvement in a broader provincial quality improvement initiative aimed at improving cirrhosis care for patients in Alberta [24]. Clinicians not directly involved in this initiative may have had different experiences in providing care to patients with cirrhosis and AUD. Second, the sample in this study was relatively homogenous, with the majority of participants being gastroenterologists/hepatologists (75%) and from urban practice zones (69%); more work is needed to understand the unique experiences of clinicians practicing in non-urban settings or in primary care or internal medicine. Third, clinicians who were interviewed were established in their professional roles; it is possible that learners (residents, fellows, etc.) have different experiences and perceptions than those who have already achieved their professional certification.

## Conclusion

This qualitative study highlights the complexities of caring for patients with concomitant cirrhosis and AUD. In caring for this unique patient population, clinicians face a myriad of challenges including limited knowledge and limited comfort with structured approaches to screening, brief intervention and treatment, inadequate access to timely resources, and competing medical sequelae that also require their attention. While identifying solutions to some of these challenges is difficult, there are tangible interventions that can be used to increase clinician knowledge on the screening, brief intervention and treatment of patients with cirrhosis and AUD. Future research should explore the effectiveness of educational interventions in improving knowledge across a range of providers, and the potential impact this has on care for patients with concomitant cirrhosis and AUD.

## Data Availability

The datasets generated and analyzed during the current study are not publicly available due individual privacy but are available from the corresponding author on reasonable request.

## Abbreviations

AUD: Alcohol use disorder
PCP: Primary Care Provider
SBIRT: Screening, Brief intervention, Referral to treatment
EASL: European Association for the Study of the Liver
